# Patterns of Use and Key Predictors for the Use of Wearable Health Care Devices by US Adults: Insights from a National Survey

**DOI:** 10.2196/22443

**Published:** 2020-10-16

**Authors:** Ranganathan Chandrasekaran, Vipanchi Katthula, Evangelos Moustakas

**Affiliations:** 1 Department of Information & Decision Sciences University of Illinois at Chicago Chicago, IL United States; 2 Middlesex University Dubai Dubai United Arab Emirates

**Keywords:** wearable healthcare devices, mobile health, HINTS, health technology adoption and use, smart wearables

## Abstract

**Background:**

Despite the growing popularity of wearable health care devices (from fitness trackes such as Fitbit to smartwatches such as Apple Watch and more sophisticated devices that can collect information on metrics such as blood pressure, glucose levels, and oxygen levels), we have a limited understanding about the actual use and key factors affecting the use of these devices by US adults.

**Objective:**

The main objective of this study was to examine the use of wearable health care devices and the key predictors of wearable use by US adults.

**Methods:**

Using a national survey of 4551 respondents, we examined the usage patterns of wearable health care devices (use of wearables, frequency of their use, and willingness to share health data from a wearable with a provider) and a set of predictors that pertain to personal demographics (age, gender, race, education, marital status, and household income), individual health (general health, presence of chronic conditions, weight perceptions, frequency of provider visits, and attitude towards exercise), and technology self-efficacy using logistic regression analysis.

**Results:**

About 30% (1266/4551) of US adults use wearable health care devices. Among the users, nearly half (47.33%) use the devices every day, with a majority (82.38% weighted) willing to share the health data from wearables with their care providers. Women (16.25%), White individuals (19.74%), adults aged 18-50 years (19.52%), those with some level of college education or college graduates (25.60%), and those with annual household incomes greater than US $75,000 (17.66%) were most likely to report using wearable health care devices. We found that the use of wearables declines with age: Adults aged >50 years were less likely to use wearables compared to those aged 18-34 years (odds ratios [OR] 0.46-0.57). Women (OR 1.26, 95% CI 0.96-1.65), White individuals (OR 1.65, 95% CI 0.97-2.79), college graduates (OR 1.05, 95% CI 0.31-3.51), and those with annual household incomes greater than US $75,000 (OR 2.6, 95% CI 1.39-4.86) were more likely to use wearables. US adults who reported feeling healthier (OR 1.17, 95% CI 0.98-1.39), were overweight (OR 1.16, 95% CI 1.06-1.27), enjoyed exercise (OR 1.23, 95% CI 1.06-1.43), and reported higher levels of technology self-efficacy (OR 1.33, 95% CI 1.21-1.46) were more likely to adopt and use wearables for tracking or monitoring their health.

**Conclusions:**

The potential of wearable health care devices is under-realized, with less than one-third of US adults actively using these devices. With only younger, healthier, wealthier, more educated, technoliterate adults using wearables, other groups have been left behind. More concentrated efforts by clinicians, device makers, and health care policy makers are needed to bridge this divide and improve the use of wearable devices among larger sections of American society.

## Introduction

### Background and Motivation

Advances in wireless sensors and digital technologies have led to a proliferation of wearable health care devices with which users can examine, monitor, and track their physiological conditions. Wearable health care devices are autonomous, noninvasive, wearable equipment with embedded sensors to collect a variety of physiological health information [1]. These devices range from the popular fitness trackers (eg, Fitbit, AppleWatch, Samsung, Galaxy Fit) that collect data on physical activities such as number of steps taken, calories burned, sleep duration, and heart rate to more sophisticated devices that can collect information on blood pressure, glucose levels, and oxygen levels. The collected health data can be transmitted to smartphones or other computer-aided applications to store and analyze to provide appropriate health interventions. Fueled by increased popularity, the use of wearables has significantly increased in recent years. According to estimates, the market for wearable health care devices in 2018 was US $24.57 billion and was slated to grow 24.7% annually to US $139.35 billion by 2026 [2].

Wearable health care devices offer several potential benefits to users. First, they offer a convenient way to monitor, store, and share health information in real-time. Second, wearables provide feedback to users to make appropriate changes to their daily routines or behavior [3,4]. Third, wearables can facilitate remote patient monitoring and provide proactive and faster data access to physicians, resulting in improved health outcomes [4,5] and reduced number of physician visits [6]. Fourth, these devices can be particularly useful for patients with chronic conditions [7], patients with cardiovascular risks [8], and elderly populations [9]. Therefore, the use of wearable devices has the potential to significantly improve health care delivery and reduce costs.

Despite potential benefits, significant challenges remain to the widespread adoption and use of wearable health care devices [10-12]. The ability of these devices to track, store, and transmit patients’ health information raises questions about data security and privacy [13-15]. In addition, the design, accuracy, and reliability of wearables have also been a major concern [16,17]. Concerns have also been raised about the accuracy of data gathered by wearables in people of color [18]. Technology acceptance of new wearable devices remains another significant barrier [19]. Despite the forecasted growth, use of these devices has reportedly slowed [20]. Market studies have pointed out a gradual decline in the use of these devices as well as abandonment within a few months of purchase [21,22].

Extant research on the use of wearables has found these devices to increase physical activity, increase energy expenditure, and reduce sedentary behavior [23]. Wearables offer an easy way to obtain large amounts of real-time health data that can be useful for clinicians [24]. Health data obtained from wearables, when combined with sophisticated machine learning algorithms, have helped develop predictive models that can greatly improve health delivery [25-27]. If effectively used, wearables can greatly help in the management of several conditions including rheumatic and musculoskeletal diseases [28,29], chronic pain management [30], and cardiovascular problems [31]. To effectively realize the potential benefits of wearable health care devices, a solid understanding of the factors related to their adoption and use is warranted. Most extant studies have largely examined the intention to adopt wearable devices [32-36] rather than the actual usage. Further, these studies have focused on a limited set of antecedents driven by the technology adoption lens. Hence, we know very little about US adults' use of wearables and key influential factors affecting usage. Addressing this gap, we build on an emergent stream of studies to examine the key factors affecting the use of wearable health care devices by US adults.

### Objectives

**Figure 1:**
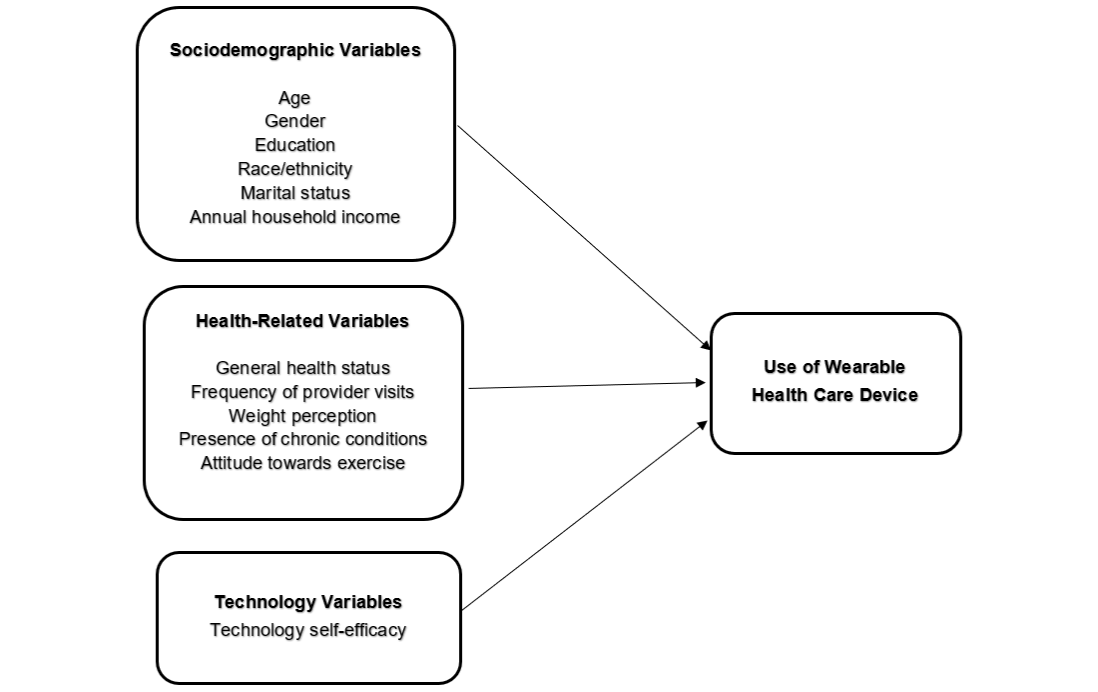
Research model with key predictors of wearable health care device use.

The main objective of this study was to examine the use of wearable health care devices and the key predictors of wearable use by US adults. We examine predictors related to individual health [37], technology self-efficacy [36,38], personal demographics [32], and attitudes towards fitness or exercise [35] as well as their associations with the use of wearable health care devices. Our research model, with all the predictor variables, is shown in Figure 1.

## Methods

### Data

The dataset for this study comes from the National Cancer Institute’s Health Information National Trends Survey (HINTS)-5, Cycle 3, with data collected from January 2019 to April 2019 through self-administered mailed questionnaires and a web-based pilot. HINTS is a nationally representative survey that includes US adults ≥18 years of age in civilian, noninstitutionalized settings. This survey collects data on US adults’ need, access, and use of information related to health and health care and the related behaviors, perceptions, and knowledge. It uses a stratified sampling method defined by areas with high concentrations of minorities and areas with low concentrations of minorities. Survey invitees for both mailed questionnaires and the web pilot involved an initial mailing of the questionnaire, followed by a reminder postcard and up to two additional mailings of the questionnaire as needed for nonrespondents. More details on the survey and survey methodology can be found elsewhere [39].

Since HINTS used probability sampling to improve representation of specific groups, our analysis applied weights to calculate US population estimates and standard errors. These sampling weights were needed to ensure unbiased estimations. Sampling weights are inverse probabilities of selection but are modified based on census or population information and further adjusted for nonresponses, so that the weighted totals of poststratification variables match the population totals. Weight adjustment accounted for nonresponse and known population totals based on the 2017 American Community Survey of the US Census Bureau on age, gender, education, marital status, race, ethnicity, and census region. We used the jackknife approach to compute replication weights [40]. Jackknife is a popular resampling approach that creates many replicate samples taken from the original sample. Each replicate sample provides an estimate of the variable of interest, and the variability among the estimates from multiple replicate samples are used to estimate the variance of the variable of interest [41].

For data analysis, we included all respondents who responded to the question about their use of an electronic health care device to track or monitor their health or physical activity in the past 12 months. Of the 5380 overall respondents, 4551 had indicated if they used (yes/no) a wearable health care device and were included in our analysis. STATA 16.1 software was used to perform the statistical analyses. We compared the demographics of all respondents with our sample that had answered the question about the use of wearable devices and did not detect any significant differences [42].

### Variables

The main variable of interest was the use of a wearable health care device. This was assessed as a binary variable (yes/no) that asked respondents to indicate their use of an electronic wearable device to monitor or track health or activity in the past 12 months. In addition, we also explored the responses about frequency of wearable health care device use in the past month (daily, almost daily, 1-2 times a week, <1 time a week, never used) and the respondents’ willingness to share the data from wearables with a provider (yes/no). Data from several survey items were included to capture survey respondents’ self-reported characteristics: sociodemographic (age, gender, race/ethnicity, education, marital status, annual household income), health-related (general health, presence of any chronic conditions, frequency of provider visits, self-perception about weight), and technology-related (technology self-efficacy) variables. General health was assessed using a question for which respondents rated their health on a Likert scale (rated as 1-5) as being poor, fair, good, very good, or excellent. Presence of chronic conditions was coded as a binary variable (0/1) if the respondents indicated they had at least one of the following conditions: diabetes or high blood sugar; high blood pressure or hypertension; heart condition such as heart attack, angina, or congestive heart failure; or a chronic lung disease such as asthma, emphysema, or chronic bronchitis. Frequency of provider visits was captured using a Likert scale based on the number of times the respondents visited a health provider in the past 12 months. Self-perception about weight was assessed on a Likert scale (rated as 1-5) as underweight, slightly underweight, just about the right weight, slightly overweight, or overweight.

As people interested in physical fitness and exercise are likely to be drawn towards a wearable health care device, another study variable included attitude towards exercise (to what extent do you enjoy exercising?). This was captured using a Likert scale (rated as 1-4), with the following options: not at all, a little, some, or a lot. Technology self-efficacy was captured using an additive score (ranging from 0 to 6 points) that was captured using questions that asked participants if they used a computer, smartphone, or electronic means for performing 6 tasks: (1) look for health information, (2) purchase medicines or vitamins online, (3) communicate with a provider using email or the internet, (4) track health care charges, (5) look up medical test results, and (6) make appointments with a provider.

### Analyses

We first conducted a descriptive analysis on our data sample. To assess the relationship between the use of wearable health care devices and sociodemographic or health-related categorical predictor variables, crosstab tables were generated and tested using the Wald chi-square test. All results were weighted to give US population level inferences using a standard weighting approach that was specifically developed for the HINTS dataset.

Given that our main variable of interest (ie, use of a wearable health care device) was a binary variable and our predictor variables were a mix of categorical and continuous variables, logistic regression analysis was chosen to assess the influence of sociodemographic variables, health-related predictors, and technology self-efficacy on the use of a wearable health care device. We computed adjusted odds ratios (ORs) and 95% CI estimates for the predictors and used a cutoff of *P*<.05 to determine the statistical significance of all our analyses. In accordance with the complex survey design, weights with jackknife replications were applied in our analyses.

## Results

**Figure 2:**
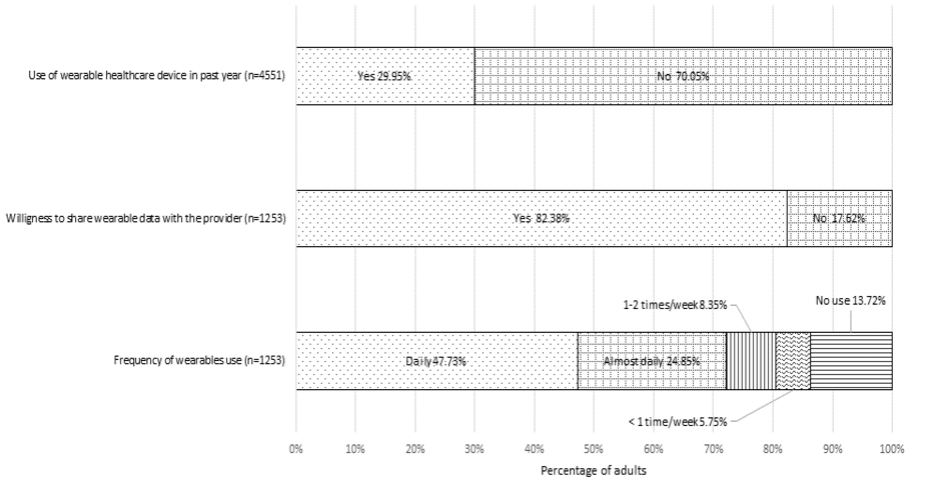
Patterns of wearable health device use by US adults.

All percentages reported in this section are the weighted values. Of the 4551 respondents who responded to the question on wearable devices, 1266 (29.95%) indicated using the device in the past 12 months. Of the adopters, 47.33% used it every day, and an additional 24.85% indicated using the device “almost every day.” Of the adopters, a majority (82.38%) reported that they were willing to share the data from wearable devices with their health care provider (Figure 2).

Table 1 presents the demographic characteristics of the respondents. Significant differences were observed between users and nonusers of wearable health care devices across different age groups, gender, education levels, and household income. Women (16.41%), White individuals (19.74%), adults aged 18-50 years (19.52%), those with some level of college education or who were college graduates (25.60%), and those with annual household incomes greater than US $75,000 (17.66%) were most likely to report using wearable health care devices. There were no significant differences based on marital status or race/ethnicity. We did not detect any significant differences in the willingness to share data from wearable devices with a provider or frequency of use of wearable devices across any of the demographic variables. Based on these results, we performed logistic regression analysis to examine how the use or nonuse of wearable devices was associated with demographic variables, health-related variables, technology self-efficacy, and attitude towards exercise.

**Table 1:** Weighted results of sociodemographic characteristics of US respondents, by use of wearable health care devices and willingness to share wearable data with a health care provider.^a^

Respondent characteristics		Use of wearable health care device in past 12 months (n=4551)				Willingness to share wearable data with health care provider (n=1253)				
		Total, %	Yes, %	No, %	*P* value^b^		Total, %	Yes, %	No, %	*P* value
Total sample, n (%)		N/A^c^	1266 (27.82)	3285 (72.18)	N/A		N/A	1013 (82.38)	240 (17.62)	N/A
**Age (years)**					<.001					.22
	18-34	26.78	10.20	16.58			34.18	30.01	4.17	
	35-49	26.50	9.32	17.18			30.85	24.71	6.14	
	50-64	30.77	7.93	22.84			25.77	20.39	5.37	
	65-74	10.47	2.00	8.47			6.65	5.12	1.53	
	≥75	5.48	0.80	4.68			2.55	2.11	0.44	
**Gender**										
	Male	49.34	13.54	35.80	.04		44.98	36.57	8.41	.55
	Female	50.66	16.41	34.25			55.02	45.81	9.21	
**Education**										
	Less than high school	4.92	0.78	4.14	<.001		2.63	2.24	0.39	.20
	High school	21.73	3.49	18.24			11.48	8.10	3.38	
	Some college	41.29	12.75	28.54			42.49	35.77	6.72	
	At least a college graduate	32.06	12.85	19.21			43.40	36.40	7.00	
**Marital status**										
	Married	69.27	20.59	48.68	.85		68.39	54.78	13.61	.03
	Other	30.73	9.32	21.41			31.61	27.86	3.75	
**Race**										
	Non-Hispanic Asian	5.84	1.76	4.09	.65		5.83	4.18	1.65	.39
	African American	10.58	2.68	7.90			8.94	7.65	1.29	
	Hispanic	16.69	5.29	11.40			17.15	13.37	3.78	
	White	63.63	19.74	43.89			64.91	55.15	9.76	
	Other	3.26	0.95	2.31			3.17	2.70	0.47	
**Household income (US $)**										
	<20,000	16.13	2.37	13.76	<.001		7.71	6.66	1.05	.73
	20,000 to <35,000	9.61	1.44	8.17			4.83	3.89	0.94	
	35,000 to <50,000	13.18	3.89	9.29			12.90	9.90	3.00	
	50,000 to <75,000	18.14	4.92	13.22			16.25	13.11	3.14	
	≥75,000	42.94	17.66	25.27			58.31	49.14	9.17	

^a^The frequency of wearable usage was omitted because no significant differences were observed based on sociodemographic characteristics.

^b^Wald chi-square test.

^c^N/A: not applicable.

The logistic regression results revealed significant associations between demographic characteristics and the use of wearable health care devices (Table 2). Individuals aged 50-64 years (OR 0.57), 65-74 years (OR 0.46), or ≥75 years (OR 0.47) were less likely to use wearable health care devices than those aged 18-34 years. Women were 1.26 times more likely to use wearable health care devices than men (OR 1.26, 95% CI 0.96-1.65). As compared to non-Hispanic Asian individuals, White individuals (OR 1.65, 95% CI 0.97-2.79) were more likely to use wearables. Individuals with higher levels of education (ie, some college education; OR 1.06, 95% CI 0.30-3.69) or who were at least college graduates (OR 1.05, 95% CI 0.31-3.51) exhibited greater likelihood to use wearables. Individuals whose annual household income was at least US $75,000 were 2.6 times more likely to use wearables (OR 2.6, 95% CI 1.39-4.86) than those with incomes less than US $20,000.

**Table 2:** The respondent characteristics that had the greatest influence in predicting the use of wearable health care devices.

Predictors		Prediction of the use of a wearable health care device in the last 12 months						
		Adjusted odds ratio^a^		95% CI		*P* value		
**Age (years)^b^**								
	35-49		0.79		0.54-1.16			.22
	50-64		0.57		0.37-0.87			<.001
	65-74		0.46		0.28-0.76			<.001
	≥75		0.47		0.24-0.89			.01
Gender^c^: Female		1.26		0.96-1.65			.01	
**Education^d^**								
	High school graduate		0.48		0.14-1.62			.14
	Some college		1.06		0.30-3.69			.04
	At least a college graduate		1.04		0.31-3.51			.05
**Race/ethnicity^e^**								
	African American		1.48		0.89-3.81			.09
	Hispanic		1.24		0.88-3.06			.12
	White		1.65		0.97-2.79			.05
	Other		1.29		0.42-4.01			.65
Marital status^f^: married		1.02		0.68-1.54			.91	
**Household income ($ US)^g^**								
	20,000 to <35,000		0.80		0.41-1.57			.51
	35,000 to <50,000		1.82		0.84-3.97			.12
	50,000 to <75,000		1.49		0.82-2.68			.18
	≥75,000		2.60		1.39-4.86			<.001
General health		1.17		0.98-1.39			.01	
Frequency of provider visits		0.97		0.89-1.05			.38	
Weight perception		1.16		1.06-1.27			<.001	
Presence of chronic conditions		0.91		0.63-1.31			.61	
Attitude towards exercise		1.23		1.06-1.43			<.001	
Technology self-efficacy		1.33		1.21-1.46			<.001	

^a^Adjusted odds ratios and 95% CIs generated from multivariate logistic regression. Model accounts for replicate weights.

^b^Reference: 18-34 years.

^c^Reference: male.

^d^Reference: less than high school.

^e^Reference: Asian.

^f^Reference: nonmarried.

^g^Reference: <20,000.

Individuals who felt their general health was better (OR 1.17, 95% CI 0.98-1.39) and those who perceived themselves to be overweight (OR 1.16, 95% CI 1.06-1.27) were more likely to adopt wearable devices. No significant differences were found based on the frequency of provider visits and for those with ≥1 chronic condition. Those who enjoyed physical activity and exercise were more likely to use health care wearables (OR 1.23, 95% CI 1.06-1.43), and individuals with higher levels of technology self-efficacy were more likely to adopt and use wearables to track or monitor their health (OR 1.33, 95% CI 1.21-1.46).

The logistic regression analysis to determine the associations between the willingness to share wearable device data with providers and all the other predictors found only two variables — race and marital status — to be significant. White adults were 3 times more likely (OR 2.87, 95% CI 1.18-7.01), and married individuals were less likely (OR 0.46, 95% CI 0.22-0.96) to share their wearable data with providers. Our examination of associations between frequency of wearable usage and the predictors did not yield any significant insights.

## Discussion

This study found wearable health care devices to be used by roughly 30% of US adults. Women, individuals with higher levels of household income, and those with higher levels of education were more likely to be wearable users. The tendency to use wearable devices seems to decline with age. US adults who consider themselves to be healthier are likely to use wearables. Other studies have also reported that healthier individuals have greater intention to adopt wearables [35]. Although individuals with chronic conditions or a higher number of provider visits have greater potential to benefit from wearables, this study did not find them to be actively using these devices. Individuals who enjoy exercise and fitness and those who are more comfortable using electronic devices exhibit a greater propensity to use wearable health care devices.

Our results indicate that only younger, healthier, wealthier, more educated, and technoliterate adults are more likely to use wearables. Younger adults are likely to be attracted to the design and aesthetic appeal of the wearable device [43], consider it “cool” [44], use it to signal their fitness intentions to peers, and also like the gamification elements such as rewards, points, and recognition of achievements awarded by the wearable devices and associated mobile apps [45-47]. Elderly adults could find wearables to be more intrusive and less comfortable [34]. Elderly adults may also not appreciate the design elements that are more geared towards younger users and would often need health professionals’ regular support and feedback to sustain the use of wearable devices [48]. Our findings imply that the design and gamification techniques in wearables that are primarily appealing to younger adults need to be customized to attract other strata of users [47]. Wearable device makers can enhance the marketability of their devices and broaden the reach of their products by coming up with devices tailored to different age groups. Our findings regarding wealthier and more educated adults to be active users of wearable technology is similar to those from other studies [37]. Educated adults are likely to be more familiar with the value they can derive from wearables. Also, given the high costs of some of the wearable devices in the market, the affordability might be better for high-income individuals [35].

Our results pertaining to the use of wearables by healthier or more health-conscious users are consistent with studies that point to the health-related motivations for using wearables such as to monitor physical activities, improve fitness, and sustain general health [49]. Individuals who set health-related goals such as weight loss and increased physical activity are likely to find wearables to be useful to monitor their progress in achieving those goals. Studies have found individuals with wellness-oriented lifestyles to engage more in preventive health behaviors like exercising regularly, and these individuals are more likely to use wearables [43]. Among other health variables, we did not find any significant association between chronic conditions and use of wearable devices. Few studies examining the use of wearable health care devices among patients with chronic conditions have reported very limited impact of these devices on disease outcomes [4]. Perhaps, such lack of tangible value for specific health conditions might discourage adults from using these devices for chronic care management purposes.

Our findings regarding the importance of technology self-efficacy validate findings from several prior studies that have examined behavioral intentions to adopt wearables from a technology acceptance perspective [35,36,50]. Individuals’ familiarity and experience with technologies such as smartphones, tablets, and other digital devices for health-related purposes would likely make them more open to using wearable health care devices. Higher technology self-efficacy can help individuals foster more positive attitudes towards smart wearables that can further augment use of these devices.

This study notes that wearable devices have not yet found widespread use among those groups that can benefit the most from them. Individuals with poor health, with chronic conditions, and who are aged can greatly benefit from tracking their physical activity and by letting their providers access the health data captured through wearables. Although our findings are consistent with other studies that have found fewer adopters of wearable devices among the senior population [51-53], they indicate a critical need to identify key barriers for low adoption of wearables among elderly US adults and work towards addressing those barriers. Factors like anxiety towards new technologies and resistance to change could impede adoption and use of wearables among elderly adults [54]. Our results have important implications for health care providers and wearable device manufacturers to educate individuals on the potential benefits of using wearables to improve health outcomes. Use cases and testimonials of wearable use by patients and elderly need to be actively publicized and promoted.

Our findings also indicate a digital divide in the US that only those with higher levels of education, household income, and technology proficiency are actively using these devices [55]. To increase patient use and engagement with digital technologies such as wearable devices, narrowing the digital divide becomes increasingly important. Making digital devices more affordable and easier to use can greatly aid in widening access to wearables and effectively using them to self-track and monitor health. This study also points to the importance of digital skills development to enable US adults to utilize wearables more effectively.

### Limitations

Little is known about the adoption and use of wearable health care devices and the predictors of adoption and use among US adults. Our work includes a broad US population and moves beyond reported intentions to use wearables to actual use of these devices. Future work should attempt to explore more predictors of the frequency of wearable usage and the actual sharing of wearable data with providers. Our study has many limitations. Although the HINTS survey used a national sample and involved stratified selection to improve the responses of subgroups, the data had responses from both mailed questionnaires and a web-based pilot, which included an incentive bonus. Web pilot responses are likely to be from adults who are more comfortable using technology and the internet, and this could possibly introduce bias. Moreover, the self-perception questions are subjective and could be interpreted differently by participants.

### Conclusions

The potential of wearable health care devices is under-realized, with less than one-third of US adults actively using these devices. With only younger, healthier, wealthier, more educated, technoliterate adults using wearables, other groups have been left behind. To effectively capitalize on the growing popularity of wearable health care devices to improve health care delivery and outcomes, more concentrated efforts by clinicians, device makers, and health care leaders are needed.

## Abbreviations

HINTS: Health Information National Trends Survey
OR: odds ratio

## References

[1] Fotiadis D, Glaros C, Likas A, Akay M (2006). Wearable Medical Devices. Wiley Encyclopedia of Biomedical Engineering.

[2] (2019). Wearable Medical Devices Market Size, Share and Industry Analysis by Product (Diagnostic & Patient Monitoring, Therapeutics), by Application (Remote Patient Monitoring and Home Healthcare, Sports and Fitness), by Distribution Channel (Retail Pharmacies, Online Pharmacies, Hypermarkets) and Regional Forecast 2019-2026. Fortune Business Insights.

[3] Finkelstein EA, Haaland BA, Bilger M, Sahasranaman A, Sloan RA, Nang EEK, Evenson KR (2016). Effectiveness of activity trackers with and without incentives to increase physical activity (TRIPPA): a randomised controlled trial. The Lancet Diabetes & Endocrinology.

[4] Jo A, Coronel BD, Coakes CE, Mainous AG (2019). Is There a Benefit to Patients Using Wearable Devices Such as Fitbit or Health Apps on Mobiles? A Systematic Review. Am J Med.

[5] Burnham JP, Lu C, Yaeger LH, Bailey TC, Kollef MH (2018). Using wearable technology to predict health outcomes: a literature review. J Am Med Inform Assoc.

[6] Landi H (2019). Patients will use health wearables to reduce trips to the doctor: survey. Fierce Healthcare.

[7] Wulfovich S, Fiordelli M, Rivas H, Concepcion W, Wac K (2019). Design Implications for Mobile Apps and Wearables Contributing to Self-Efficacy of Patients With Chronic Conditions. Front Psychol.

[8] Kańtoch E (2018). Recognition of Sedentary Behavior by Machine Learning Analysis of Wearable Sensors during Activities of Daily Living for Telemedical Assessment of Cardiovascular Risk. Sensors (Basel).

[9] Stavropoulos TG, Papastergiou A, Mpaltadoros L, Nikolopoulos S, Kompatsiaris I (2020). IoT Wearable Sensors and Devices in Elderly Care: A Literature Review. Sensors (Basel).

[10] Patel M, Asch D, Volpp K (2015). Wearable devices as facilitators, not drivers, of health behavior change. JAMA.

[11] Xue Y (2019). A review on intelligent wearables: Uses and risks. Human Behav and Emerg Tech.

[12] Piwek L, Ellis DA, Andrews S, Joinson A (2016). The Rise of Consumer Health Wearables: Promises and Barriers. PLoS Med.

[13] Amyx S (2017). Privacy Dangers of Wearables and the Internet of Things. Identity Theft: Breakthroughs in Research and Practice.

[14] Perez A, Zeadally S (2018). Privacy Issues and Solutions for Consumer Wearables. IT Prof.

[15] Li H, Wu J, Gao Y, Shi Y (2016). Examining individuals' adoption of healthcare wearable devices: An empirical study from privacy calculus perspective. Int J Med Inform.

[16] Loncar-Turukalo T, Zdravevski E, Machado da Silva J, Chouvarda I, Trajkovik V (2019). Literature on Wearable Technology for Connected Health: Scoping Review of Research Trends, Advances, and Barriers. J Med Internet Res.

[17] Chuah S, Rauschnabel P, Krey N, Nguyen B, Ramayah T, Lade S (2016). Wearable technologies: The role of usefulness and visibility in smartwatch adoption. Computers in Human Behavior.

[18] Hailu R (2019). Fitbits and other wearables may not accurately track heart rates in people of color. STAT Internet.

[19] Sergueeva K, Shaw N, Lee SH (2019). Understanding the barriers and factors associated with consumer adoption of wearable technology devices in managing personal health. Can J Adm Sci.

[20] Nieroda M, Mrad M, Solomon M (2018). How do consumers think about hybrid products? Computer wearables have an identity problem. Journal of Business Research.

[21] (2016). Gartner survey shows wearable devices need to be more useful. Gartner.

[22] Ledger D, McCaffrey D (2014). Inside wearables: How the science of human behavior change offers the secret to long-term engagement. Endeavour Partners.

[23] Brickwood K, Watson G, O'Brien J, Williams AD (2019). Consumer-Based Wearable Activity Trackers Increase Physical Activity Participation: Systematic Review and Meta-Analysis. JMIR Mhealth Uhealth.

[24] Wu J, Li H, Cheng S, Lin Z (2016). The promising future of healthcare services: When big data analytics meets wearable technology. Information & Management.

[25] Bini S, Shah R, Bendich I, Patterson J, Hwang K, Zaid M (2019). Machine Learning Algorithms Can Use Wearable Sensor Data to Accurately Predict Six-Week Patient-Reported Outcome Scores Following Joint Replacement in a Prospective Trial. J Arthroplasty.

[26] Nweke H, Teh Y, Al-garadi M, Alo U (2018). Deep learning algorithms for human activity recognition using mobile and wearable sensor networks: State of the art and research challenges. Expert Systems with Applications.

[27] Meng Y, Speier W, Shufelt C, Joung S, E Van Eyk J, Bairey Merz CN, Lopez M, Spiegel B, Arnold C (2020). A Machine Learning Approach to Classifying Self-Reported Health Status in a Cohort of Patients With Heart Disease Using Activity Tracker Data. IEEE J. Biomed. Health Inform.

[28] Davergne T, Pallot A, Dechartres A, Fautrel B, Gossec L (2019). Use of Wearable Activity Trackers to Improve Physical Activity Behavior in Patients With Rheumatic and Musculoskeletal Diseases: A Systematic Review and Meta-Analysis. Arthritis Care Res (Hoboken).

[29] Leese J, Macdonald G, Tran B, Wong R, Backman C, Townsend A, Davis A, Jones CA, Gromala D, Avina-Zubieta JA, Hoens A, Li L (2019). Using Physical Activity Trackers in Arthritis Self-Management: A Qualitative Study of Patient and Rehabilitation Professional Perspectives. Arthritis Care Res (Hoboken).

[30] Janevic MR, Shute V, Murphy SL, Piette JD (2020). Acceptability and Effects of Commercially Available Activity Trackers for Chronic Pain Management Among Older African American Adults. Pain Med.

[31] Leong I (2018). Exercise: Wearable activity trackers in cardiovascular research. Nat Rev Endocrinol.

[32] Zhang M, Luo M, Nie R, Zhang Y (2017). Technical attributes, health attribute, consumer attributes and their roles in adoption intention of healthcare wearable technology. Int J Med Inform.

[33] Park E (2020). User acceptance of smart wearable devices: An expectation-confirmation model approach. Telematics and Informatics.

[34] Papa A, Mital M, Pisano P, Del Giudice M (2020). E-health and wellbeing monitoring using smart healthcare devices: An empirical investigation. Technological Forecasting and Social Change.

[35] Lee S, Lee K (2018). Factors that influence an individual's intention to adopt a wearable healthcare device: The case of a wearable fitness tracker. Technological Forecasting and Social Change.

[36] Wang H, Tao D, Yu N, Qu X (2020). Understanding consumer acceptance of healthcare wearable devices: An integrated model of UTAUT and TTF. Int J Med Inform.

[37] Li J, Ma Q, Chan AH, Man SS (2019). Health monitoring through wearable technologies for older adults: Smart wearables acceptance model. Appl Ergon.

[38] Abouzahra M, Ghasemaghaei M (2020). The antecedents and results of seniors’ use of activity tracking wearable devices. Health Policy and Technology.

[39] Methodological Reports of HINTS: Health Information National Trends Survey. National Cancer Insitute.

[40] Kolenikov S (2018). Resampling Variance Estimation for Complex Survey Data. The Stata Journal.

[41] Lavrakas PJ (2008). Jackknife Variance Estimation. Encyclopedia of survey research methods.

[42] (2020). HINTS 5 Cycle 3 Public Codebook.

[43] Dehghani M, Kim K, Dangelico R (2018). Will smartwatches last? factors contributing to intention to keep using smart wearable technology. Telematics and Informatics.

[44] Kim J, Park E (2019). Beyond coolness: Predicting the technology adoption of interactive wearable devices. Journal of Retailing and Consumer Services.

[45] Ridgers N, McNarry M, Mackintosh K (2016). Feasibility and Effectiveness of Using Wearable Activity Trackers in Youth: A Systematic Review. JMIR Mhealth Uhealth.

[46] Schaffarczyk L, Ilhan A (2019). Healthier Life and More Fun? Users' Motivations to Apply Activity Tracking Technology and the Impact of Gamification. https://link.springer.com/chapter/10.1007/978-3-030-21905-5_10.

[47] Spil T, Sunyaev A, Thiebes S, Van Baalen R (2017). The Adoption of Wearables for a Healthy Lifestyle: Can Gamification Help?. https://aisel.aisnet.org/hicss-50/hc/apps_for_health_management/7/.

[48] Brickwood K, Williams AD, Watson G, O'Brien J (2020). Older adults' experiences of using a wearable activity tracker with health professional feedback over a 12-month randomised controlled trial. Digit Health.

[49] Maher C, Ryan J, Ambrosi C, Edney S (2017). Users' experiences of wearable activity trackers: a cross-sectional study. BMC Public Health.

[50] Lunney A, Cunningham N, Eastin M (2016). Wearable fitness technology: A structural investigation into acceptance and perceived fitness outcomes. Computers in Human Behavior.

[51] Kekade S, Hseieh C, Islam MM, Atique S, Mohammed Khalfan A, Li Y, Abdul SS (2018). The usefulness and actual use of wearable devices among the elderly population. Comput Methods Programs Biomed.

[52] Pobiruchin M, Suleder J, Zowalla R, Wiesner M (2017). Accuracy and Adoption of Wearable Technology Used by Active Citizens: A Marathon Event Field Study. JMIR Mhealth Uhealth.

[53] Mercer K, Giangregorio L, Schneider E, Chilana P, Li M, Grindrod K (2016). Acceptance of Commercially Available Wearable Activity Trackers Among Adults Aged Over 50 and With Chronic Illness: A Mixed-Methods Evaluation. JMIR Mhealth Uhealth.

[54] Talukder M, Sorwar G, Bao Y, Ahmed J, Palash M (2020). Predicting antecedents of wearable healthcare technology acceptance by elderly: A combined SEM-Neural Network approach. Technological Forecasting and Social Change.

[55] Kontos E, Blake KD, Chou WS, Prestin A (2014). Predictors of eHealth usage: insights on the digital divide from the Health Information National Trends Survey 2012. J Med Internet Res.

